# Influence of Pear Variety and Drying Methods on the Quality of Dried Fruit

**DOI:** 10.3390/molecules25215146

**Published:** 2020-11-05

**Authors:** Agata Marzec, Hanna Kowalska, Jolanta Kowalska, Ewa Domian, Andrzej Lenart

**Affiliations:** 1 Department of Food Engineering and Process Management, Institute of Food Science, Warsaw University of Life Sciences—SGGW, 159c Nowoursynowska St., 02-776 Warsaw, Poland; hanna_kowalska@sggw.edu.pl (H.K.); ewa_domian@sggw.edu.pl (E.D.); andrzej_lenart@sggw.edu.pl (A.L.); 2 Division of Food Quality Evaluation, Institute of Food Science, Warsaw University of Life Sciences—SGGW, 159c Nowoursynowska St., 02-776 Warsaw, Poland; jolanta_kowalska@sggw.edu.pl

**Keywords:** dried pear, microwave-convective drying (hybrid), texture, acoustic emission and mechanical properties, sensory analysis

## Abstract

In this study, the impacts of two different pear cultivars, “Conference” and “Alexander Lucas”, on the kinetics and the final quality of samples dried by convection (CD) and microwave-convection (MCD) methods, were investigated. The quality of dried material was evaluated by the analysis of water activity, porosity, color, acoustic emission (AE) and mechanical and sensory properties. The required drying time to obtain 0.2 kg H_2_O/kg dry solid (d.s.) was longer for “Conference” than “Alexander Lucas” and was 20 min by CD and 5 min by MCD. The pear cultivar, in conjunction with the drying method (CD or MCD), affected the number of AE events and the work of breaking. The CD pear of the “Conference” cultivar was characterized by higher force, higher breaking work and stronger AE relative to the CD pear of the “Alexander Lucas” cultivar. There were no differences in taste or overall quality, but the hardness was higher for the CD “Conference” pear. A principal component analysis showed that panelists preferred dried fruit with good taste and overall quality but lower hardness. A positive correlation was found between the number of acoustic events and sensory hardness; thus, an acoustic method can be useful for effectively evaluating the texture of dried pears. These results show that the dried pear slices that generated fewer AE events upon breaking were perceived as better by the panelists.

## 1. Introduction

Snacks made of fruit and vegetables are increasingly popular and defined by textural characteristics such as crispness and crunchiness, which reflect their quality and even their freshness [1]. Crispy/crunchy snacks break relatively easily and produce a sharp sound [2,3]. One example is dried apples, which are high in nutritional value and suitable for direct consumption. Drying pears makes it possible to provide alternative products to consumers.

In research on the quality of dried fruit, a homogeneous raw material is used, so pre-treatment conditions and drying process parameters are optimized to obtain a product with the best quality. In industrial practice, the raw material varies, which further affects the resulting dried product. Therefore, there is a need to closely study the relationships between the parameters of the raw material used and the quality of the finished dried material so that it is possible to predict the quality characteristics of the product, which, in turn, will ensure that the end product is consistently of a good quality. The most important varieties of pears cultivated in Poland are “Conference” and “Alexander Lucas”. These varieties differ in their content of readily hydrolyzed sugars [4]. Studies have evaluated the drying characteristics of different varieties of pears, such as “Ankara” [5], “Santa maria” [6], “Blanquilla” [7], “Deveci” [6,8], “Williams” [9], “D. Joaquina” [10,11], “Rocha” [12,13] and “Conference” [14,15]. The main ingredient in pears is water (about 85%), and the remainder is a dry substance, which mainly contains carbohydrate (14%), fiber (2%), protein (0.3%) and fat (0.1%). Pears are a good source of vitamins and minerals [6,11,16]. Drying causes the concentration of ingredients, as well as chemical and physical changes in the material [17,18]. Enzymatic and non-enzymatic browning reactions produce flavor components and compounds that modify the taste and texture of dried fruit and additionally lead to the loss of nutritional value [19]. Under the influence of temperature and oxidation of the raw material, the product darkens due to the action of polyphenol oxidase and non-enzymatic reactions.

The most commonly used method for drying fruit is convective drying. This process and the resulting products are reviewed in [18,20]. The long drying time at relatively high temperatures during the falling rate periods of convective drying often leads to undesirable thermal degradation of the products [21]. Hybrid drying is used to obtain dried fruit with higher quality [17,22]. An example of such drying is microwave-convective or ultrasonic-assisted drying. The number of possible combinations of different techniques at different stages of the drying is enormous. This has resulted in many new hybrid technology developments [7,23].

Dried pears have been obtained by different drying methods: sun [24], convective [6,8,10,12,15], microwave-vacuum [25], osmotic pre-treated convective [7,9,14], osmo-vacuum [26], ultrasound-assisted infrared and freeze-drying [27,28]. There is no information in the literature on microwave-convective drying of the pears. Microwave-convective drying is gaining interest due to its unique volumetric heating capability and ability to significantly reduce drying time and improve quality [20]. The use of microwaves in convective drying can save significant amounts of energy [23]. For strawberries, the total energy consumption of the convective-continuous microwave drying method was one-tenth that of convective drying [23].

The impact of drying methods and conditions on pears can be deduced from the color, shrinkage and density of the dried fruit and, above all, from the analysis of the dried fruit texture, mainly by assessing its crispness/crunchiness [25].

Using a trained panel to evaluate sensory textures, including crispness, is time-consuming and expensive; thus, it is important to find a method that can facilitate the efficient assessment of the texture of dried fruit [29]. The instrumental measurement of texture will facilitate the selection of the drying method to produce dried pears with the best textural properties, which are highly desired by consumers. Crispness is perceived by the senses and is caused by the mechanical shredding of the product, accompanied by the emission of sound [30,31].

According to Andreani et al. [2], acoustical and mechanical instrumental tests can help to predict and compare the sensory crispness of food products. Acoustic emission (AE) is particularly useful, as its parameters strongly correlate with sensory results [2,31,32]. The acoustic methods of food texture testing use the registration of elastic waves that result from dynamic, local changes in the structure of the analyzed product. Waves that form inside or outside of the tested material are caused by mechanical stress [2,33,34].

The aim of this study was to evaluate the impacts of the variety and drying method on the kinetics of drying pears as well as on the water activity, density, color, acoustic and mechanical properties and sensory properties of the dried material. An additional objective was to determine whether the acoustic emission method and mechanical test are suitable for evaluating the texture of dried pears and comparable to a sensory assessment.

## 2. Results and Discussion

### 2.1. Drying Kinetics, Water Activity and Density Analysis

**Figure 1:**
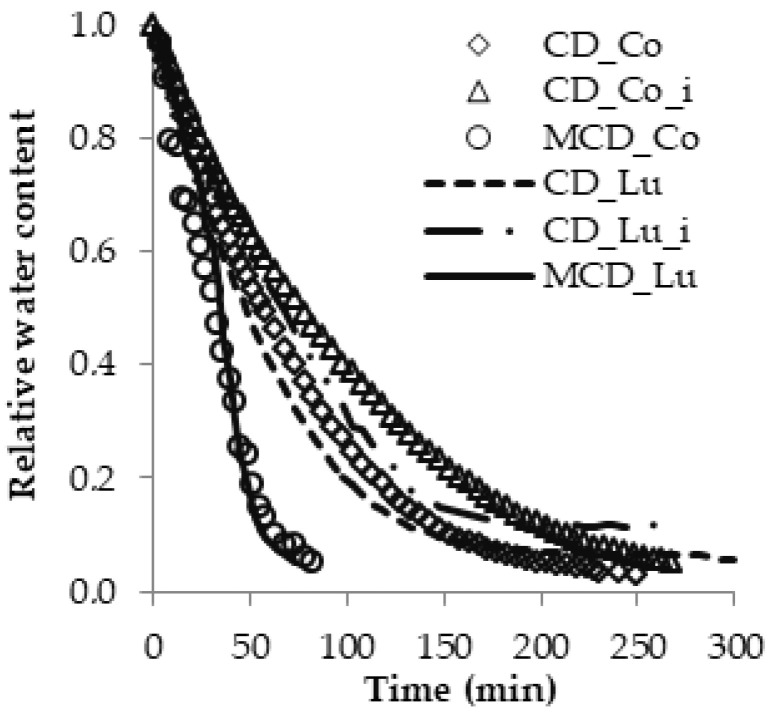
Pear drying kinetics (“Conference” (Co) and ”Alexander Lucas” (Lu) were dried with a convection method (CD) at 60 °C and microwave-convection method (MCD) at 40 °C, power 300 W; i—immersed in citron acid).

The pears varieties “Conference” (Co) and “Alexander Lucas” (Lu) show similar drying kinetics (Figure 1). In a typical drying process, moisture from the solid material first evaporates from the moisture layer on the surface and then continuously decreases until the water inside of the material moves outward by diffusion processes [5,35] The drying time required to reach a similar relative water content depended on the pear cultivar and drying method (Figure 1). Comparing the convection drying of pear slices of both cultivars, it can be concluded that “Lu” pears dried faster than “Co”. The rate of water removal during drying is influenced by the high sugar content in pears, which can range from 50 to 65 kg/100 kg product. In the drying process, the sugar concentration increases with the evaporation of water, which, combined with the shrinkage of the fruit, results in additional resistance to moisture transfer [36]. In our experiment, “Co” pears had a higher degrees Brix than “Lu” pears, which means that they contained more sugars, and this could be the reason for slower drying. However, the time required to obtain the same final moisture content was longer for “Lu” than in the case of “Co”. This paradoxical conclusion follows from the fact that both cultivars had different initial water content. This means that in order to obtain a final water content of 0.20 kg H_2_O/kg dry solid (d.s.), more water needs to be removed from “Lu” pears than from “Co” pears.

Compared with convection drying, microwave-convection drying resulted in smaller differences in the kinetics between pear cultivars. The combination of microwaves and convection significantly reduced the drying time. A relative water content of 0.08 occurred after 85 min in “Co” pears and after 80 min in “Lu”. The reduction in drying time was caused by the increase in vapor pressure within the dried samples when using microwave heating, enabling faster migration of moisture to the product surface. The microwave application can enhance heat and mass transfer, which can be attributed to the properties of microwave heating because microwaves can penetrate the internal medium in the form of electromagnetic waves [23,29]. These results are comparable to those reported in previous studies for drying fruit such as pears, strawberries, hawthorns and *Sorbus domestica* [7,8,13,29,37,38].

Immersion of pears in a citric acid (CA) solution before convection (CD) in order to reduce the color change significantly affected the drying kinetics (Figure 1). Citric acid slowed the drying process. A relative water content of 0.08 of the “Co” variety was obtained after 227 min and was further reduced to 0.06 after 250 min. In contrast, “Lu” achieved a relative water content of 0.11, and this value did not change with prolonged drying. Öztekin and Sacilik [5] studied “Ankara” pears and found that pear slices treated with citric acid required shorter drying times than untreated samples. Similarly, Doymaz [39] found that immersion for 2 min in a solution of 0.5% citric acid at room temperature accelerated the drying process of apples. This is not in line with the results obtained in our work. It is possible that our results were affected by the short time spent dipping pear slices and then draining them with tissue paper. Although pectin in the cell walls of fruit generally degrades more in an acidic environment, the concentration of citric acid might not have been high enough in this case to exhibit a clear effect. Pectins in plant cell walls vary in their solubility [40]. In the case of fruit, especially pears, the sugar concentrations (on a wet basis) are relatively high and increase very significantly as water evaporates, offering, combined with fruit shrinkage, additional resistance to moisture transfer from the fruit throughout the drying process [36]. Therefore, the drying of pears becomes critically dependent on the temperature as well as the water and sugar concentrations inside the fruit during the drying process.

In addition to the drying method, the pear variety was found to significantly affect the water content and water activity of the dried samples (*p* < 0.001) (Table 1).

The “Co” variety dried by CD and microwave-convection (MCD) had higher water activity than “Lu”, with values below 0.17 and 0.14, respectively. The immersion of pears in a citric acid solution did not significantly affect the water activity of the dried materials (Table 1). The type and intensity of the deteriorative reactions that may occur in food materials strongly depend on water activity. Microbial growth, enzymatic and non-enzymatic activities and other deteriorative reactions in foods are predicted according to water activity. Each product has a critical activity that corresponds to the monolayer capacity, which is determined from sorption isotherms [41]. The best food stability is observed in the monolayer moisture content. Djendoubi Mrad et al. [41] showed that the stability of dried fruit could be determined on the basis of water activity and glass transition temperature. The glass transition concept suggests that products are stable at or below the corresponding glass transition temperature. The glass transition temperature depends on the water content. Food physicochemical changes and textural properties such as hardness, crispness, collapse, amorphous-to-crystalline transformations and the rate of non-enzymatic browning not only depend on the monolayer value but also correlate with the glass transition temperature through plasticization by water or temperature. The authors determined the glass transition temperature as a function of the water content of pears and connected this with isotherm sorption. The glass transition temperature of pears decreased with the decrease in solids content, confirming a strong plasticizing effect. Critical water activities corresponding to the investigated temperature range (30–60 °C) varied from 0.002 to 0.080 for the pears, and critical moisture content varied from 0.020 to 0.087 g/g dry matter. Therefore, Djendoubi Mrad et al. [41] recommended that water activity in dried pears be below 0.08 to increase the stability of the material during storage, but for dried fruits, a water activity lower than 0.2 is sufficient.

The chemical and physical transformations that occur during the drying process influence the end-product structure and texture, which have a decisive influence on its attractiveness to the consumer. The material density changes constitute a parameter that defines the structure. Experimental data on the particle density of the pears are shown in Table 1. The particle density varies with the pear cultivar and does not depend on the drying method. This corresponds to the data published by Krokida [42], who reported that different drying methods did not affect the particle density of bananas, apples, carrots or potatoes. CD pears of the “Co” variety had significantly lower apparent density than dried “Lu”. In our study, the drying method did not affect the apparent density of dried pears (Table 1). Some authors have reported that the method and parameters of drying affected the density of the dried material [10,14,42]. Djendoubi Mrad et al. [14] found that during CD at air temperatures of 30–70 °C, “Co” pears had different densities when the moisture of the dried fruit was lower than 0.5 kg/kg d.s.

Fruits are composed of cellular tissues and, therefore, may be regarded as peculiar porous systems [14]. Dried slices of “Co” pears were characterized by a lower porosity compared with “Lu”. In both varieties, the MCD process resulted in greater porosity than the CD method. This reflects the different structures of the tested materials. Fluctuations in the porosity of individual types of dried fruit are the result of the employed drying method and its parameters, but they mainly depend on the chemical composition and the initial water content, the structure of the material and the mechanisms of moisture transport [43,44].

### 2.2. Color Analysis

Table 2 shows the effect of the pear cultivar and the drying method on the color parameters of the dried fruit. Fresh “Co” pears were slightly darker than “Lu”, but they had a significantly lower value of the a* parameter and a nearly two-fold higher b* parameter. Both varieties had different values of chroma (C*), hue angle (h*) and yellowness index (YI) (Table 2). The color parameters of dried pears differed from those of fresh fruit (Table 2). The color change in dried pears of the “Co” and “Lu” varieties shows that severe browning occurred. The browning of the fruit is most often attributed to phenolic compound oxidation and caramelization [19,20,29].

The influence of the variety on the lightness (L*) parameter value of the dried fruit was not statistically significant. In contrast, the values of C* and YI, which are color brightness indices for materials, were higher for dried “Co” pears compared with “Lu” (Table 2). Dried pears of the “Co” variety had a YI of about 15% higher than that of the “Lu” variety. According to Duan et al. [29], the YI is used to evaluate total degradation from all processes (including quantification of scorching, soiling and general product degradation by light, chemical exposure and processing) with a single value. High C* and YI values imply that drying “Co” pears led to more physiological changes, e.g., browning and nutrient degradation [29]. The observed changes could be caused by the long drying time of fruit, which results in longer exposure to oxygen. The chemical composition of pears is also important. “Co” pears had a higher tissue extract content than “Lu”. The immersion of the fruit in the citric acid solution caused a significant color change, but only in pears of the “Co” variety (Table 2). Citric acid lowers the pH, thereby reducing the rate of enzymatic browning. This was effective with “Co” pears. These differences may result from the activity of polyphenol oxidase, organic acid content, sugar concentration and pH [14].

The drying process influenced the color of the dried fruit. Physical and chemical changes that occur during drying are caused by pigment concentration due to water loss, dye degradation, enzymatic processes and gas exchange [20,29]. The drying method had a significant effect on the total color change (ΔE) of the material, hue angle (h*) and the yellowness index (YI) (Table 2). Dried MCD pears retained more color than dried CD pears (Table 2). The use of microwaves reduces the drying time and, consequently, changes in the color of the material.

A significant interaction effect of the variety and the drying method on the L*, a* and h* color parameters was demonstrated (Table 2). Drying CD pears of the “Co” variety led to a darker color compared with the “Lu” variety. However, the use of MCD had the opposite effect. Pear slices of the “Co” variety dried by MCD were lighter than the “Lu” variety.

### 2.3. Acoustic and Mechanical Properties

**Figure 2:**
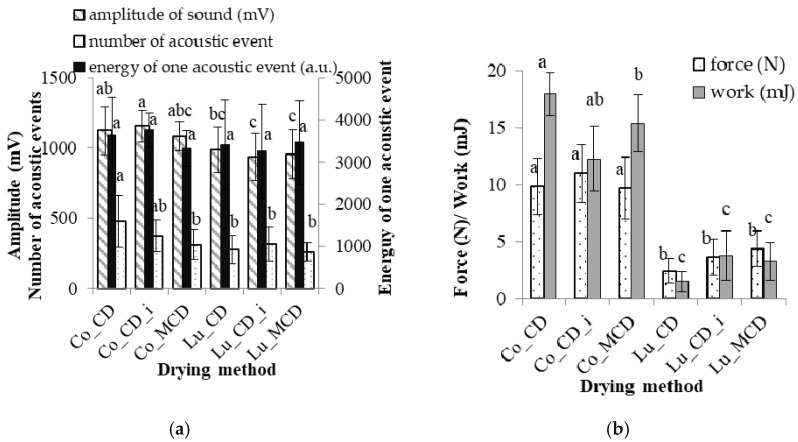
Pear drying: (**a**) acoustic and (**b**) mechanical parameters (“Conference” (Co) and ”Alexander Lucas” (Lu) dried with a convection method (CD) at 60 °C and microwave-convection method (MCD) at 40 °C, power 300 W; i—immersed). a, b, c—homogeneous groups of varieties and drying methods of pears.

The analysis of variance test results for the pears are summarized in Table 1. The pear cultivar affected the sound amplitude and the number of acoustic events of the dried fruit. Convection-dried pears of the “Co” variety generated a significantly higher amplitude of sound and a greater number of acoustic events than “Lu” (Figure 2a). The energy of one acoustic event was not dependent on the pear variety.

Furthermore, the pear variety influenced the force and breaking work of dried fruit (Table 1, Figure 2b). These mechanical parameters were approximately two times higher for dried “Co” pears than those in the case of “Lu” (Figure 2b). The obtained results show that, depending on the variety, the mechanical properties of dried fruit demonstrate a high degree of variability. In materials with porous and crisp structures, the breaking force oscillates due to the breaking of the walls and structure disintegration [45].

The differences between the pear cultivars used in the present study are the result of their differing chemical compositions, including initial water content and tissue extract of 13.4 °Brix and 9.7 °Brix in “Co” and “Lu” pears, respectively. Pears of both varieties contain sugars (mainly fructose) and fiber [4]. The removal of water during the drying process causes the concentration of the solution components. Low-molecular-weight substances may crystallize or form an amorphous state. Bonazzi and Dumoulin [43] stated that dry products are predominantly obtained in a glassy amorphous form from most of the common drying processes. Substances in an amorphous state have the structure of a liquid but the properties of a solid. Dehydrated food materials often remain in the metastable, non-equilibrium amorphous state for extended periods, but they can also exhibit time-dependent changes [43]. The dried pears had low water activity and were tested immediately after drying. It is possible that the differences in the chemical composition and structure between the two pear varieties had an impact on the acoustic and mechanical properties. In addition, the significantly higher density of the “Co” pear relative to “Lu” could have been the reason for its harder texture (higher force, breaking work, amplitude of sound and number of acoustic events).

The immersion of pears in a citric acid solution before CD did not have a significant influence on the acoustic or mechanical properties of either pear variety (Figure 2a,b). The analysis of the effect of the drying method (convection at 60 °C and microwave-convection at 40 °C and 300 W) showed that it had a significant correlation only with the number of acoustic events of the “Co” pear variety (Figure 2a). The drying methods used in our work did not affect the acoustic or mechanical parameters of “Lu” pears (Figure 2a,b). Moreover, a significant interaction of the drying method with the pear variety was found (Table 1). There was no indication that the described features affected other AE parameters, as presented in Figure 2a. Thus, the authors of the paper infer that the number of acoustic events can be regarded as a measure of crispness in the evaluation of dried fruit. Previous researchers have investigated the textural properties of crispy products through acoustic and mechanical testing and have shown that the number of acoustic events was correlated with sensory crispness [2,31,32]. Crispness is one of the most important parameters of fruit snacks that are produced by drying. A crispy texture is the result of a porous structure formed by air cavities surrounded by brittle structures [46]. High temperature and the velocity of airflow during CD cause the surface layer to close up, forming a barrier that impedes water diffusion from inside towards the outside of the dried material. Convection drying causes high shrinkage and cell deformation. The thermal stresses that are generated cause internal structural damage such as cracks and changes in material geometry. Therefore, the dried pear had a high number of acoustic events. In contrast, MCD causes the effect of strong absorption, allowing water to heat up quickly inside the dried material. This decreases the drying time, contraction and structural defects.

**Figure 3:**
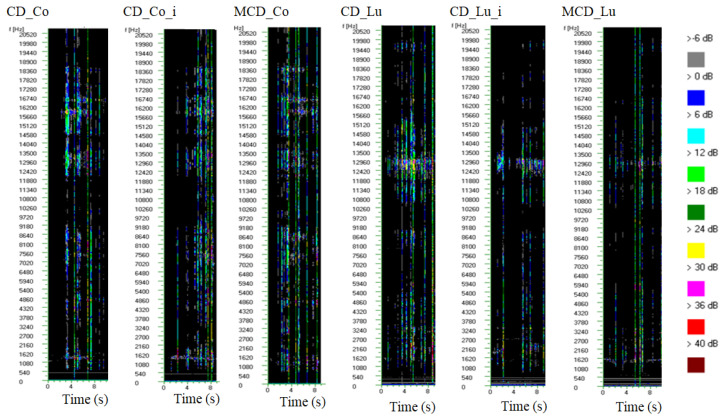
Spectral sound characteristics of dried pears. The analyzed frequency band (1–20 kHz) is presented on the vertical axis, and the horizontal axis denotes time in seconds.

The above-described dependencies were also confirmed by the spectral characteristics of the analyzed dried pears (Figure 3). The spectral characteristics are presented in the form of “acoustograms”. Colors in the acoustograms represent the power of the signal in time–frequency coordinates [31,47]. The frequency of sound is useful for differentiating between crisp and crunchy products. Crunchy products are characterized by a frequency lower than 2 kHz. On the other hand, crispy products emit higher-frequency sounds above 5 kHz [48]. In our study, the sound frequencies produced upon breaking the dried pears were different between the two varieties (Figure 3). Pears of the “Co” variety produced dominant frequencies in the ranges of 1–9 kHz and 12–18 kHz (Figure 3). Dried pears of the “Lu” variety emitted sounds of frequencies in the ranges of 1–5 kHz, 8–9 kHz and 10–15 kHz during the total breaking time (Figure 3). The drying method did not cause changes in the sound frequencies of the analyzed samples. However, the power of the signal was higher when breaking CD pears compared with MCD pears (Figure 3). According to Lewicki et al. [34], the spectral characteristics of AE signals can be a useful tool for testing cereal products with different moisture contents. Our study confirms that spectral characteristics can also be used to evaluate the texture of dried fruits. Dried “Co” pears were louder and harder than “Lu”. Moreover, it was shown that the cultivar of the pear had a significant effect on the hardness, and thus, the texture of the final product was determined by the cultivar but not the drying method (CD and MCD).

### 2.4. Sensory Properties

**Figure 4:**
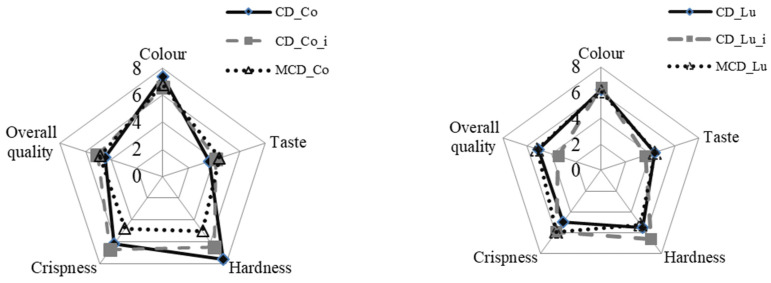
Sensory properties of pears (“Conference” (Co) and ”Alexander Lucas” (Lu)) dried with a convection method (CD) at 60 °C and microwave-convection method (MCD) at 40 °C, power 300 W; i—immersed).

A significant effect of the variety on the sensory characteristics of dried pears was observed (Figure 4). The assessed sensory hardness of CD pears of the “Co” variety was higher than that in the case of “Lu”. However, “Co” dried fruit obtained lower scores in the sensory evaluation for taste and overall quality, and it was characterized by the darkest color (Figure 4). In the sensory evaluation, the crispness and hardness of convection-dried pears initially immersed in CA solution were higher, but the taste was evaluated as worse in comparison with pears that had not been immersed in CA (Figure 4). No statistically significant differences in overall impression were found between drying treatments within varieties. Although the fruit dried by the MCD method received higher marks in the sensory evaluation for taste and overall quality and was characterized by lower hardness and less crispness than fruit dried using CD only, statistical differences were not found (Figure 4). The drying method affected the hardness of the “Co” variety only (Table 1). Convection-dried “Co” pears were significantly harder than those dried by microwave-convection. The literature suggests that because the drying time of MCD is shortened compared with CD, it results in greater preservation of aromatic components and biologically active substances and improves the sensory characteristics [20,29].

### 2.5. A Complex Evaluation of the Dried Fruit Texture

**Figure 5:**
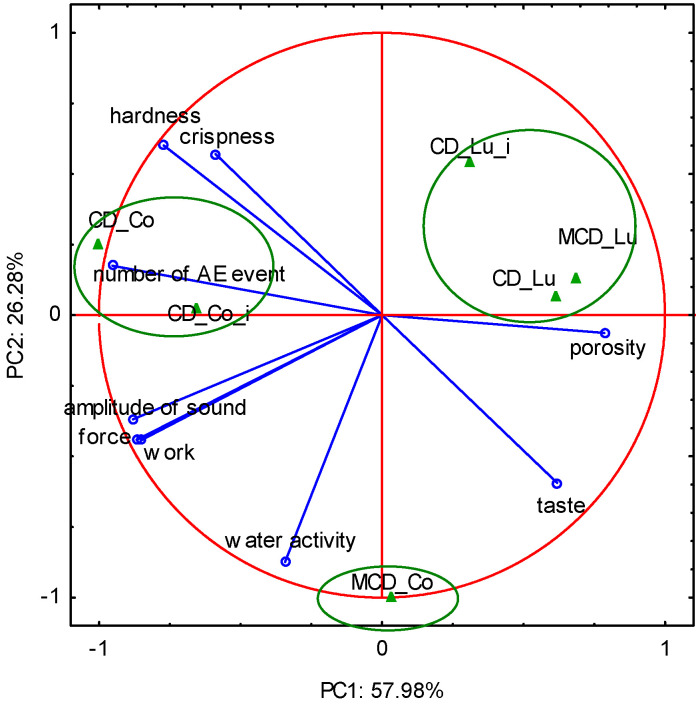
First two components of PCA for acoustic, mechanical and sensory parameters of dried pears (Co—“Conference”; Lu—“Alexander Lucas”; CD—convective drying; MCD—microwave-convective drying; i—immersed).

With the aim of determining the most suitable parameters for the description of the dried pear texture, a principal component analysis (PCA) was conducted. In the PCA, the results related to the water activity, porosity and the acoustic, mechanical and sensory properties of the two pear cultivars dried by different methods were incorporated into two new, uncorrelated variables termed “principal components” (PC1 and PC2). The relations between parameters and PCs were interpreted according to the correlations between them. The results show that the variety of pear had an influence on the textural properties of the dried fruit. Two components, PC1 and PC2, explained 84.26% of the variability of the textural properties of dried pears (Figure 5), with a simultaneous loss of 15.7% of the information. Most of the parameters were characterized by a high loading factor that corresponded to PC1. The first component (PC1) was negatively correlated with the acoustic parameters (number of AE events, amplitude of sound) as well as the mechanical parameters (force and work of breaking) and sensory characteristics (hardness, crispness). PC1 was positively correlated with the taste and porosity of dried pears. The second principal component (PC2) was correlated with water activity (Figure 5). Correlations between acoustic and sensory properties were observed. The number of AE events had a positive correlation with hardness and crispness and a negative correlation with porosity. The mechanical parameters (force and breaking work) were not related to sensory attributes. Furthermore, correlations between water activity and acoustic, mechanical and sensory properties were not observed. Sensory analysis showed that CD pears of the “Co” variety (with and without pre-immersion in CA solution) were described as hard and crisp, and hardness correlated with the number of AE events. The results obtained revealed that the CD (with and without pre-immersion in CA solution) and MCD pears of the “Lu” variety were located in the positive area of the first dimension, which was mainly defined by porosity. “Co” pears dried by the microwave-convection method resulted in products with high water activity and low hardness and crispness.

Similar to our work, a previous study revealed correlations between the acoustic and sensory parameters of dried apples [32]. The implementation of the acoustic technique in agriculture and food engineering could be considered a viable technology in quality assessment [2,30].

## 3. Materials and Methods

### 3.1. Materials

Pear (*Pyrus communis* L.) was purchased from a domestic market (Poland). During the experiment, the fruit was stored in a climate chamber at 1 °C and about 90% humidity. Experiments were carried out in October and November.

The water content of fresh pear was 4.81 kg H_2_O/kg dry solid (d.s.) for the “Conference” variety (Co) and 6.25 kg H_2_O/kg dry solid (d.s.) for the “Alexander Lucas” variety (Lu); their refractive indices were 13.4 °Brix and 9.7 °Brix, respectively. Seed nests were cut from the fruit, and then the pears were mechanically cut into 3 mm transverse slices. Afterwards, the total quantity was divided into two batches. The first part was immersed in 1% (1 g/100 g H_2_O) citric acid (CA) for 1 min at 25 °C, and then it was washed with distilled water and slightly drained by using filtrate paper. The second part did not undergo any pre-treatment. The material prepared in this way was dried using various methods. Pears were dried in a single layer to a final moisture content of about 0.20 kg H_2_O/kg d.s. Moisture content was calculated from the weight difference between the fresh and the dried sample and expressed as g per g of the dry solid (d.s.) [32]. Dried pears were stored for 4 weeks at controlled humidity (0%) and temperature (25 °C) for further quality comparison.

### 3.2. Drying Methods

Two drying methods were used in the experiment: convection (CD) and microwave-convection (MCD). In order to obtain dried material with high quality, preliminary tests were carried out, and the results were used to select the temperature and microwave power of the drying process. Approximately 200 g of sliced pear was dried in each experiment. CD was carried out at three different temperatures (60, 70 and 80 °C). For MCD, microwave power (200 and 300 W) was tested at 30 and 40 °C. The drying parameters for our experiment were chosen based on the sensory features of dried pears. Additionally, according to the literature, at 60 °C, convection drying results in optimal vitamin C preservation [14]. The convective drying method was carried out at a temperature of 60 °C and with an airflow of 1.5 m·s^−1^ using a convective laboratory air dryer. The dryer works as an open-loop system. The mass of the samples was continually monitored during drying. The change in sample mass during drying was recorded by the computer every 3 min. The CD experiments continued until a constant mass was obtained.

Pear slices were dried in the laboratory microwave-convection (MCD) dryer PROMIS-μLAB (“PROMIS”, Wroclaw, Poland), which allowed the regulation and measurement of temperature, microwave power and mass change [32]. The slices of pears were placed on the rotating sieve. Two hundred grams of fresh pear was used for each drying cycle. Tests were conducted at a temperature of 40 °C with an air velocity of 1.5 m·s^−1^ using a microwave power of 300 W. The microwave generator was equipped with a magnetron continuous wave (2450 MHz). Drying was controlled through a computer with a specialized program that allowed the parameters of the drying process to be set. The drying parameters, such as temperature, microwave power, the final mass of pears and time of the drying cycle, were established. After one drying cycle (3 min), the control program stopped the fan and switched off the generator of the microwaves. At that moment (3 s), the mass and the temperature of the material were measured. The measurement results were sent to the computer. After that, the process of microwave drying was restarted. When the material reached a constant mass, the computer control program ended the microwave drying process. All drying experiments were performed twice.

### 3.3. Density and Water Activity

The apparent density (ρ) was measured in 3 repetitions using a helium pycnometer from Stereopycnometer (Quantochrome Instruments, Boynton Beach, FL).

The particle density (ρ_p_) was calculated using the formula given by Krokida [42]:

(1) $$\rho_{p}=\frac{1+W}{\frac{1}{\rho_{\mathrm{SS}}}+\frac{W}{\rho_{W}}}$$

where W is water content (kg⋅kg^-1^), and ρ_w_ is the density of water (1020 kg·m^−3^). The density of the pear dry mass (ρ_ss_) was calculated from the following equation:

(2) $$\rho_{\mathrm{ss}}=\frac{\sum_{i=1}^{n}x_{i}}{\sum_{i=1}^{n}\frac{x_{i}}{\rho_{i}}}$$

where x_i_ and ρ_i_ are the content and density of the component and pear dry mass, respectively: 2% and 1400 (kg·m^−3^) protein, 0.5% and 930 (kg·m^−3^) fat, 91.4% and 1580 (kg·m^−3^) sugars and 2% and 1440 (kg·m^−3^) fiber.

Total porosity (ε) was calculated from the following equation [43]:

(3) $$\varepsilon =1-\frac{\rho_{p}}{\rho }$$

Water activity was determined in 3 repetitions using the Hygroscope DT2 device (Rotronic, Switzerland).

### 3.4. Color Measurements

The color of the pear was measured before and after drying using a colorimeter (CHROMA METER CR-300, Konica Minolta) at three points on the samples. The color parameters were measured, including lightness (L*), with 100 being very white and 0 being dark; the a* value, which represents greenness (−60) to redness (+60), and the b* value, which represents blueness (−60) to yellowness (+60), were measured and used for the calculation of total color difference (ΔE) [29]. The values of chroma (C*), hue angle (h*) and yellowness index (YI) were calculated using the formula given by Duan et al. [29].

### 3.5. Acoustic and Mechanical Properties

The acoustic emission (AE) was registered with a contact method using the 4381 sensor (Brüel & Kjær, Narum, Denmark) in a three-point test when pear slices underwent breakage in the Zwick 1445 (ZWICK GmbH, Ulm, Germany) at a constant deformation speed of 0.5 mm/s. The distance between supports was 24 mm. The sensor was connected to the AE signal amplifier with a 2 m cable. The acoustic emission signal was amplified (40 dB) in the low-noise amplifier and digitalized using an Adlink Technology Inc. type 9112 (Adlink Technology Inc., Taipei, Taiwan) analog-digital conversion sound card with a sampling frequency of 44.1 kHz [32]. Details of the measurement, calculation and definition of acoustic descriptors were presented by Lewicki at al. [34]. The acoustic descriptors were the amplitude of sound (μV), number of AE events and energy of one AE event (a.u.), which were calculated using the Calculate_44kHz_auto program (Warsaw, Poland) [34]. The acoustic descriptors obtained by the AE method were extracted at a discrimination level of 1000 mV. The acoustic signal was also analyzed in the frequency domain, which was used to obtain the power spectrum characteristics.

The following mechanical parameters were calculated: force and breaking work. Breaking work was calculated as the area under the deformation curve of dried pear slices. Fifteen repetitions were carried out in these analyses [32].

### 3.6. Sensory Properties

The sensory evaluation was conducted by 15 trained panelists, 10 women and 5 men (all between 20 and 24 years of age), in laboratory conditions. Prior to testing, three training sessions were held to familiarize the panelists with the products and with the attributes that were selected, as described in the PN-ISO standard [49]. The following characteristics were selected as the most important in determining the quality and attractiveness of dried pears: crispness (defined as the noise level at which a product breaks when biting it with molar teeth), hardness, taste, color and overall quality of dried fruit. The tests were conducted according to the ISO 4121:2003 standard [49] using the method of random sampling. The sensory analysis included the following categories, with scores based on a hedonic scale from 0 to 10: crispness (quiet: 0; loud: 10), hardness (gentle: 0; very hard: 10); color (creamy yellow: 0; dark-honey-cream color: 10); pear taste (imperceptible: 0; very intense: 10); overall quality of dehydrated food (bad: 0; very good: 10). Samples were coded by 2-digit random numbers in random order. Water (room temperature) was used as a neutralizer between the evaluation of different samples.

### 3.7. Statistical Analysis

An analysis of variance (ANOVA) was conducted using Statistica 12 PL, and significant differences in average values were determined using Duncan’s Multiple Range test at a significance level of *p* < 0.05. For variables that did not follow a normal distribution, the division into homogeneous groups was performed using the non-parametric multiple comparison test (Kruskal–Wallis test). Principal Component Analysis (PCA) was also performed.

## 4. Conclusions

From the results obtained in this study, it can be concluded that the water activity (a_w_) and density are higher in the dried pear slices of the “Conference” variety than “Alexander Lucas”. The drying method also affected a_w_, but it did not influence the density of the dried fruit. The pear cultivar significantly affected the drying time. The “Conference” pear took longer to dry than “Alexander Lucas”. The “Conference” pear was more prone to change color during drying than the “Alexander Lucas” variety.

The results also show that the variety of pears affects the texture of the dried. The “Conference” and “Alexander Lucas” varieties affected both the acoustic and mechanical properties and sensory hardness of dried fruit but did not influence the following sensory characteristics: color, taste, crispness and overall quality. The drying method (CD or MCD) had a significant influence only on the number of acoustic events and sensory hardness of the “Conference” and “Alexander Lucas” dried pears. The drying method affected the texture of the dried fruit within the individual pear varieties. Therefore, in order to obtain a homogeneous, dry, hard and crunchy texture, it is recommended to use specific varieties of pears.

Convection-dried pears with or without pre-immersion in a citric acid solution were characterized by higher force, higher breaking work and stronger texture as a consequence of their low density. Water activity and porosity did not significantly affect the sensory perception of dried pears.

The acoustic emission parameter (number of acoustic events) of pears dried by the different methods was correlated with the sensory hardness of the dried fruit. Therefore, the acoustic method can be recommended for the measurement of dried texture and for a more rapid assessment of the texture of dried pears in comparison with sensory evaluation.

## Figures and Tables

**Table 1:** Effect of the variety and the drying method on the physicochemical parameters of dried pears (each value is presented as mean ± SD) (“Conference” (Co) and ”Alexander Lucas” (Lu) were dried with a convection method (CD) at 60 °C and microwave-convection method (MCD) at 40 °C, power 300 W; i—immersed in citron acid).

Sample	Moisture (g/g)	Water Activity	Drying Time	Apparent Density (g/cm^3^)	Particle Density (g/cm^3^)	Porosity (%)
CD_Co	2.25 ± 0.40 ^c^	0.171 ±0.001 ^e^	250	1.198 ± 0.051 ^a^	1.129 ± 0.032	5.7 ± 0.7 ^a,b^
CD_Co_i	0.83 ± 0.25 ^a^	0.139 ± 0.005 ^c^	267	1.317 ± 0.029 ^e^	1.235 ± 0.182	6.1 ± 0.7 ^b^
MCD_Co	2.75 ± 0.12 ^d^	0.222 ± 0.005 ^f^	80	1.236 ± 0.050 ^b^	1.113 ± 0.105	10.0 ± 0.5 ^a^
CD_Lu	1.55 ± 0.05 ^b^	0.147 ± 0.008 ^b,c^	298	1.267 ± 0.017 ^c^	1.161 ± 0.551	8.2 ± 0.5 ^c^
CD_Lu_i	0.85 ± 0.15 ^a^	0.152 ± 0.009 ^d^	300	1.400 ± 0.022 ^e^	1.227 ± 0.125	12.3 ± 0.7 ^d^
MCD_Lu	1.35 ± 0.10 ^b^	0.145 ± 0.006 ^b^	85	1.359 ± 0.021 ^d^	1.176 ± 0.095	13.1 ± 0.1 ^d^
**Level of Significance**						
Variety	<0.001 *	<0.001 *	-	0.015 *	<0.001 *	<0.001 *
The method of drying	<0.001 *	<0.001 *	-	0.062	0.110	<0.001 *
Variety X method drying	<0.001 *	<0.001 *	-	0.400	0.001 *	0.008 *

^a, b, c, d, e, f^—homogeneous group; * 5% significance.

**Table 2:** Effects of the pear cultivar and drying method on measured parameters.

	L*	a*	b*	C*	h*	ΔE	YI
Co_fresh	71.05 ± 1.01 ^x^	−1.74 ± 0.05 ^y^	14.97 ± 0.47 ^x^	15.08 ± 0.59 ^x^	96.61 ± 0.69 ^y^	-	15.80 ± 0.91
CD_Co	68.66 ± 1.03 ^a,b^	6.92± 1.02 ^b,c^	36.73 ± 1.24 ^a^	37.38 ± 1.41 ^c^	79.39± 1.17 ^b^	23.55 ± 1.63 ^b^	76.45 ± 3.73 ^c^
CD_Co_i	67.08 ± 1.51 ^a^	9.31± 2.53 ^c^	33.57 ± 1.10 ^b^	34.89 ± 1.41 ^b,c^	74.61 ± 3.93 ^a^	24.06 ± 3.27 ^b^	71.49 ± 5.68 ^b,c^
MCD_Co	72.64 ± 0.76 ^c^	2.68 ± 0.72 ^a^	33.31 ± 1.11 ^b^	33.42 ± 1.16 ^a,b^	85.47 ± 1.08 ^c^	18.94 ± 1.2 2 ^a^	65.51 ± 6.77 ^a,b^
Lu_fresh	72.97 ± 1.05 ^x^	−1.22 ± 1.23 ^x^	8.08 ± 0.49 ^y^	8.17 ± 0.59 ^y^	98.58 ± 0.28 ^x^	-	30.10 ± 0.78
CD_Lu	69.03 ± 0.31 ^a,b^	5.47 ± 0.36 ^a,b^	30.83 ± 0.85 ^a^	31.31 ± 0.87 ^a^	79.98± 0.53 ^b^	24.04 ± 0.87 ^b^	63.80 ± 1.77 ^a,b^
CD_Lu_i	70.60 ± 2.20 ^b,c^	5.01 ± 1.81 ^a,b^	30.68 ± 1.81 ^a^	31.11 ± 2.09 ^a^	80.88 ± 2.67 ^b^	23.64 ± 2.49 ^b^	62.19 ± 5.68 ^a^
MCD_Lu	69.52 ± 3.28 ^a,b,c^	5.43 ± 2.25 ^a,b^	30.09 ± 1.88 ^a^	30.61 ± 2.28 ^a^	79.98 ± 3.39 ^b^	23.41 ± 2.92 ^b^	62.04 ± 6.75 ^a^
**Level of Significance**							
Variety	0.529	0.941	<0.001 *	<0.001 *	0.474	0.049 *	0.003 *
Drying of method	0.028 *	0.009 *	0.150	0.072	0.012 *	0.024 *	0.041 *
Variety X method drying	0.015 *	0.006 *	0.486	0.234	0.007 *	0.052	0.095

^x, y^—homogeneous groups of fresh pears; ^a, b, c^—homogeneous groups of varieties and drying methods of pears; * 5% significance.

## Abbreviations

Co	Pear of the “Conference” variety
Lu	Pear of the “Alexander Lucas” variety
CD	Convective drying method
MCD	Microwave-convective drying method
CA	Citric acid
i	Immersion in CA
C*	Chroma
ΔE	Chroma
h*	Hue angle
YI	Yellowness index
